# A hypergravity environment increases chloroplast size, photosynthesis, and plant growth in the moss *Physcomitrella patens*

**DOI:** 10.1007/s10265-016-0879-z

**Published:** 2016-11-28

**Authors:** Kaori Takemura, Hiroyuki Kamachi, Atsushi Kume, Tomomichi Fujita, Ichirou Karahara, Yuko T. Hanba

**Affiliations:** 1 Department of Applied Biology, Kyoto Institute of Technology, Matsugasaki, Sakyo-ku, Kyoto, 606-8585 Japan; 2 Graduate School of Science and Engineering, University of Toyama, 3190 Gofuku, Toyama, 930-8555 Japan; 3 Faculty of Agriculture, Kyushu University, 6-10-1 Hakozaki, Higashi-ku, Fukuoka, 812-8581 Japan; 4 Faculty of Science, Hokkaido University, Kita-ku, Sapporo, 060-0810 Japan

**Keywords:** Bryophyte, Cell wall, CO_2_ diffusion, Evolution, Gametophore

## Abstract

The physiological and anatomical responses of bryophytes to altered gravity conditions will provide crucial information for estimating how plant physiological traits have evolved to adapt to significant increases in the effects of gravity in land plant history. We quantified changes in plant growth and photosynthesis in the model plant of mosses, *Physcomitrella patens*, grown under a hypergravity environment for 25 days or 8 weeks using a custom-built centrifuge equipped with a lighting system. This is the first study to examine the response of bryophytes to hypergravity conditions. Canopy-based plant growth was significantly increased at 10×*g*, and was strongly affected by increases in plant numbers. Rhizoid lengths for individual gametophores were significantly increased at 10×*g*. Chloroplast diameters (major axis) and thicknesses (minor axis) in the leaves of *P. patens* were also increased at 10×*g*. The area-based photosynthesis rate of *P. patens* was also enhanced at 10×*g*. Increases in shoot numbers and chloroplast sizes may elevate the area-based photosynthesis rate under hypergravity conditions. We observed a decrease in leaf cell wall thickness under hypergravity conditions, which is in contrast to previous findings obtained using angiosperms. Since mosses including *P. patens* live in dense populations, an increase in canopy-based plant numbers may be effective to enhance the toughness of the population, and, thus, represents an effective adaptation strategy to a hypergravity environment for *P. patens*.

## Introduction

In the evolutionary history of plants, bryophytes have been suggested to be among the oldest plant groups to expand their habitats from aquatic to land environments (Rubinstein et al. 2010). Bryophytes may have been subjected to marked changes in many environments including buoyancy, light, temperature, and water conditions. Among these environmental changes, the effects of gravity on land may undoubtedly have induced alterations in the physiology, anatomy, and growth of bryophytes, extrapolated from previous findings obtained from vascular plants. Gravity influences the physiological processes of vascular plants including long-distance water transport (Lambers et al. 2008), leaf gas exchange (Hirai and Kitaya 2009), cell proliferation (Matía et al. 2010), and cell wall rigidity (Hoson and Wakabayashi 2015) and, thus, plant growth (Hangarter 1997). Nevertheless, few studies have investigated the gravitational responses of bryophytes even though gravitropism has been extensively examined (Banbury 1962; Cove and Quatrano 2006; Martin et al. 2009; Repp et al. 2004; Schwuchow et al. 2002); only one study showed the significant effect of microgravity on the growth pattern of the dark-grown protonemata of the moss *Ceratodon purpureus* (Kern et al. 2005). Physiological and anatomical analyses have yet to be performed on the gravitational responses of bryophytes. A comparison of the physiological and anatomical responses to changes in gravity in bryophytes and vascular plants will provide crucial information for estimating how plant physiological traits have evolved to adapt to significant changes in gravity in land plant history.

Among bryophyte species, the moss *Physcomitrella patens* is suitable for physiological and anatomical analyses of responses to gravity. An analysis of the anatomy and growth of *P. patens* may be easily performed from the cellular level because it has a simple plant form with a single stem of <100 cells per its diameter as well as single cell-layered rhizoid and leaves (phyllids). Furthermore, it is possible to perform a genetic analysis of the responses of *P. patens* to gravity because complete genome sequencing has already finished for this moss (Rensing et al. 2008).

The creation of an altered gravity environment is essential for analyzing plant responses to increases in gravity. Hypergravity environments produced by centrifugation have provided a practical experimental system for angiosperms (Hoson et al. 1996; Manzano et al. 2012; Nava et al. 2015; Soga et al. 2015; Tamaoki et al. 2014; Waldron and Brett 1990; Zhang et al. 2013). However, these experiments were often performed under dark conditions with strong hypergravity (>30×*g*), and did not involve bryophytes. Therefore, we designed a new centrifuge equipped with a lighting system, which enables plant growth under moderate hypergravity environments (<10×*g*) with irradiance, in order to investigate the mechanisms by which *P. patens* grows under long-term hypergravity conditions under light conditions.

Although it is important to note that differences existed between our experimental conditions (in the light) and those of previous studies (in the dark), several alterations are expected in the growth and anatomy of *P. patens* grown under moderate hypergravity. Short-term strong hypergravity under dark conditions (>30×*g*) was previously reported to result in significant reductions in shoot elongation for various angiosperms including pea, cress, maize, azuki bean, and *Arabidopsis thaliana* (Hoson et al. 1996; Soga et al. 1999a, b, 2001; Tamaoki et al. 2006; Waldron and Brett 1990; Zhang et al. 2013). Although a previous study using moderate hypergravity (2×*g* and 6×*g*) for four days under dark conditions reported no effects on seedling elongation in *A. thaliana* (Manzano et al. 2012), the microgravity environment created by four days of spaceflight under dark conditions induced enhancements in the shoot and root growth of *A. thaliana* seedlings (Matía et al. 2010). Based on these findings, we expect long-term moderate hypergravity to induce reductions in the shoot and rhizoid growth of *P. patens.* Therefore, alterations in the lateral plant growth of *P. patens* stems may be possible. A short-term 300×*g* environment under dark conditions increased the cross-sectional area of the seedlings of azuki bean (Soga et al. 2006) and *A. thaliana* (Tamaoki et al. 2011). Similar alterations are expected in *P. patens* stems.

Although changes in the shoot growth of angiosperms by hypergravity are partly attributable to the reduced translocation of organic solutes (Zhang et al. 2013), major factors for assessing growth alterations by hypergravity are changes in plant cell walls, including extensibility (Nakabayashi et al. 2006) affected by enhanced lignification (Wakabayashi et al. 2009). These changes in cell walls may induce reductions in CO_2_ diffusion through plant cells, resulting in a lower photosynthesis rate in plants. The cell wall is one of the major factors affecting CO_2_ diffusion conductance inside the leaves of angiosperms (Terashima et al. 2011) and ferns (Carriquí et al. 2015), with a thicker cell wall inhibiting CO_2_ diffusion inside leaves within angiosperms (Kogami et al. 2001) and fern species (Tosens et al. 2016). Although the relationship between cell wall thickness and CO_2_ diffusion inside plants has not yet been examined for bryophytes, alterations in cell walls induced by hypergravity are expected to reduce CO_2_ diffusion in bryophytes as well as vascular plants, and, thus, decrease photosynthesis rates.

Other physiological alterations in the leaves of bryophytes related to the photosynthesis rate may be possible with hypergravity. Exposure to short-term strong hypergravity (500-2000×*g*) under dark conditions was previously reported to induce reductions in the chlorophyll content and photosynthesis rate in wheat seedlings grown for five days under 1×*g* (Vidyasagar et al. 2014), which suggests that some alterations in chloroplast functions and impairments in photosynthetic biochemistry are caused by hypergravity.

Anatomical and physiological alterations are expected in response to hypergravity in *P. patens*. The aims of this study are (1) to quantify changes in the plant growth of *P. patens* grown under long-term hypergravity created by a centrifuge with irradiance, (2) to assess anatomical determinants for these alterations in the growth of *P. patens*, and (3) to analyze the post-effects of hypergravity on the photosynthesis rate of *P. patens* in relation to changes in plant growth and anatomy.

## Materials and methods

### Plant material and cultivation under hypergravity conditions

Gametophores of *P. patens* Bruch & Schimp subsp. patens (Ashton and Cove 1977) were maintained in a growth chamber at 25 °C under continuous white light. The medium used was BCD medium (Nishiyama et al. 2000) solidified with 0.8% (w/v) Difco Bacto Agar. One- to two-month-old gametophores cut into 3-mm lengths from the shoot apex were placed on agar medium in 5-cm Petri dishes and then incubated for 3–5 days under continuous white light to immobilize them on agar medium. Petri dishes were sealed with surgical tape (type 21 N, NICHIBAN Co., Ltd., Tokyo, Japan) to prevent rapid desiccation. After being incubated, the gametophores were grown under 1×*g* or hypergravity conditions of 2.3, 4.0, 7.1, and 10×*g* at 25 °C for the designated times.

**Fig. 1:**
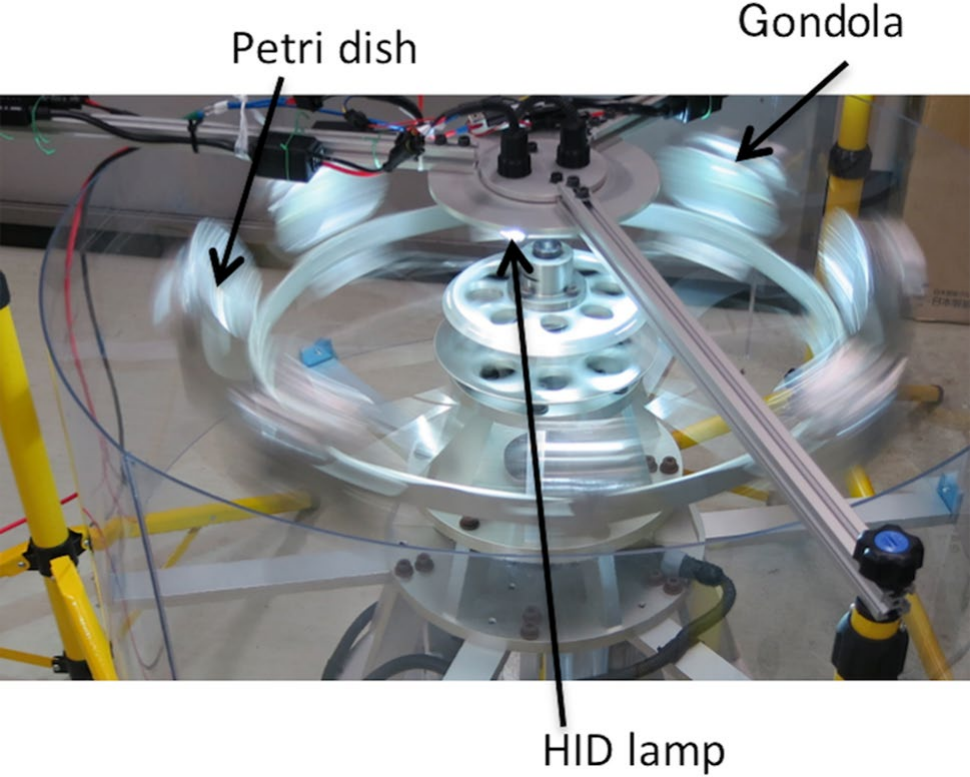
A photo of a custom-built centrifuge (MIJ-17, Environmental Measurement Japan, Co., Ltd, Fukuoka, Japan) equipped with the High Intensity Discharge lamp (HID, 35 W-6000 K-H4, Lexno, China)

In hypergravity experiments, we used a custom-built centrifuge (Fig. 1, MIJ-17, Environmental Measurement Japan, Co., Ltd, Fukuoka, Japan) equipped with a High Intensity Discharge lamp (HID, 35 W-6000 K-H4, Lexno, China) of 30 µmol m^−2^ s^−1^ of PPFD, which enables long-term plant cultivation under moderate hypergravity conditions. Regarding the 1×*g* controls, Petri dishes were placed in the same temperature-controlled room and grown under the same HID with the same PPFD. The effective arm length of MIJ-17 was 33 cm, at which the gravity gradient along the horizontal plane and the vertical direction was negligible (e.g. Morita et al. 2015). A long-term growth experiment using the centrifuge with Petri dishes was not problematic for the growth of *P. patens* because its growth rate was so slow that it only reached <2 cm in height even after 8 weeks of growth. Any small vibrations generated by the centrifuge equipment appeared to have had negligible effects on the growth of *P. patens* in Petri dishes because (1) different levels of hypergravity, which induced different levels of vibration, resulted in similar plant growth, and (2) we obtained similar growth responses using two different centrifuges with different levels of vibration. We consider the CO_2_ concentration in the Petri dishes to have been similar between the hypergravity and 1×*g* environments because we confirmed that the diffusive conductance of water vapor of the Petri dishes was similar. In order to prevent drying, we added medium diluted 2-fold once a week.

### Plant growth

We examined the effects of hypergravity on plant growth before the detailed anatomical analysis and gas exchange measurements. Gametophores grown at 2.3, 4.0, and 7.1×*g* and at 1×*g* as a control for 25 days were used to measure shoot length, shoot diameter at the middle of the shoot, rhizoid length for each gametophore (*n* = 10–14), and the number of gametophores in the canopy (e.g., population of gametophores in a Petri dish, *n* = 5–6). The ratios of these parameters to those for the 1×*g* controls were calculated. The minimum hypergravity condition without a loss in light intensity in the centrifuge MIJ-17 was 2.3×*g*. Therefore, we selected 7.1×*g* as approximately 3-fold hypergravity of the minimum condition, and 4.0×*g* as the middle of these logarithmic values.

Gametophores grown at 10×*g* and 1×*g* for 8 weeks were used in a detailed analysis of plant growth. The length and dry mass of shoots and rhizoids were measured for each gametophore (*n* = 75–76) and each canopy (*n* = 6–7), respectively. The numbers of gametophores for each canopy were measured (*n* = 6–7). In order to measure stem and leaf traits, each gametophore was mounted fresh and photographed (PowerShot SX210, Canon, Tokyo, Japan) to obtain stem and leaf images. Ten to fifteen fresh gametophores and five leaves from four fresh gametophores (*n* = 20) were used to measure stem (caulid) diameter and leaf length (between the leaf tip and base), respectively, at two positions of the gametophores; 3 mm from the top (‘top’) and 3 mm from the base (‘base’).

### Microscopy

Gametophores grown at 10×*g* and 1×*g* for 8 weeks were subjected to an anatomical analysis. Prior to observations, samples were settled in a dark refrigerator for a few days. Light micrographs were taken using a microscope (BX51-33, OLYMPUS, Tokyo, Japan). Images were digitally recorded with a CCD camera (VB-7010, KEYENCE, Osaka, Japan) and analyzed using ImageJ software (http://imagej.nih.gov/ij/). The numbers of chloroplasts in each cell in fresh leaves (phyllids) were measured for 4–5 leaf cells at the central part of the lamina from the middle part of the shoot for thirteen gametophores (*n* = 54–56) at ×400 magnification.

In subsequent morphological and anatomical analyses, measurements were performed at two positions in gametophores; 3 mm from the top (top) and 3 mm from the base (base). In the analyses of the density of cells, cell sizes, and cell wall thickness, stem or leaf sections were fixed in 2.5% glutaraldehyde in 0.2 M sodium phosphate buffer (pH 7.4) for 20 min, post-fixed in 1% OsO_4_ solution at 4 °C for 3 h in darkness, and then embedded in Spurr’s resin (Low Viscosity Resin kit, TAAB, Aldermaston, UK). Longitudinal and transverse sections of stems, and transverse sections of leaves with 1000-nm-thick were stained with 1% toluidine blue solution. The density of stem cells was measured from transverse sections of the stems (*n* = 4–6) by counting cell numbers in these sections and then dividing them by the areas of the sections. The sizes of stem epidermal cells, which are suggested to be determinants for stem elongation (Kutschera and Niklas 2007; Savaldi-Goldstein and Chory 2008), were measured for 6–7 epidermal cells from longitudinal sections of the stems of gametophores (*n* = 20–22) at ×200 magnification. Lamina thickness was measured at the 5th cell from the costa of the leaf, lamina cell diameter was averaged from the 1st to 5th cell from the costa (*n* = 3–11), and lamina cell numbers from the costa to the margin of the lamina were measured for 3–6 leaves using the transverse sections of leaves.

Chloroplasts of *P. patens* were lens-shaped, and the diameters (major axis) and thicknesses (minor axis) of 20 chloroplasts were measured from the 1st to 10th cell from the costa of a leaf at the top and base from 3 gametophores (*n* = 60), and for 15 chloroplasts from the epidermal cells of the stems of 3 gametophores (*n* = 45) at ×400 magnification. Quantification was performed for chloroplasts facing the air and attached to cell walls. The cell wall thicknesses of stems and leaves were measured for five epidermal cells of stems and the 1st to 5th cell from the margin of the lamina, respectively, with three parts of a cell being selected for measurements (*n* = 45) at ×1000 magnification. The ultrastructure of chloroplasts in leaves from the middle part of the gametophore was observed using a transmission electron microscope (JEM-1220, JOEL, Tokyo, Japan), on which ultrathin 70-nm-thick sections were mounted on copper grids, stained in 2% uranyl acetate for 15 min, and then in Reynold’s lead citrate for 5 min.

### Measurement of photosynthesis

Photosynthesis measurements for *P. patens* were performed 2–3 days after the hypergravity experiments had finished using a system constructed by the Kyoto Institute of Technology based on previous studies (Kawase et al. 2013; Tosens et al. 2015). The measurement of photosynthesis in gametophores was performed according to the standard methods of previous studies (Meyer et al. 2008; Waite and Sack 2010). Rhizoids were removed from gametophores and then saturated with distilled water by complete immersion. Excess external water was removed from the gametophore surface using filter paper, giving a fully turgid mass. We measured photosynthesis in *P. patens* at a fully turgid mass because it is close to the optimal tissue water content for photosynthesis (Meyer et al. 2008); we confirmed this by performing photosynthesis measurements under saturated irradiance (500 µmol m^−2^ s^−1^) for a drying cycle of gametophores (4–5 h, data not shown). In photosynthesis measurements, gametophores were placed in an acrylic chamber (12 cm × 10 cm × 2 cm), with the temperature of plant materials being monitored using a thermocouple. Temperature was adjusted to 25 °C, the flow rate was 300-350 ml min^−1^, relative humidity was approximately 90%, and the CO_2_ concentration was set at 400 µmol mol^−1^. A red and blue LED (red: blue 8:1, LEDRB-630DL, Opto Code Corp., Tokyo, Japan) was used as a light source. We measured boundary layer conductance in accordance with area by obtaining a calibration curve using filter papers with different areas (data not shown). A fan placed inside the chamber mixed the air completely. The response of the photosynthesis rate (net CO_2_ assimilation rate) to changing irradiance was obtained at various photosynthetic photon flux densities (PPFD) from 0 to 800 µmol m^−2^ s^−1^ at a fully turgid mass. At least 5 min was needed at each PPFD to stabilize the photosynthesis rate. The photosynthesis rate per area was calculated from the projected canopy area of a moss canopy using a scanner.

### Statistical analysis

Differences between means were tested by Welch’s *t* test for independent samples, performed using R software (Rcmdr package, ver3.2.4). The effects of gravity [1×*g* vs. hypergravity (2.3, 4.0, 7.1, and 10×*g*)] and positions of the stems (top vs. base) were tested by Welch’s *t*-test.

## Results

**Fig. 2:**
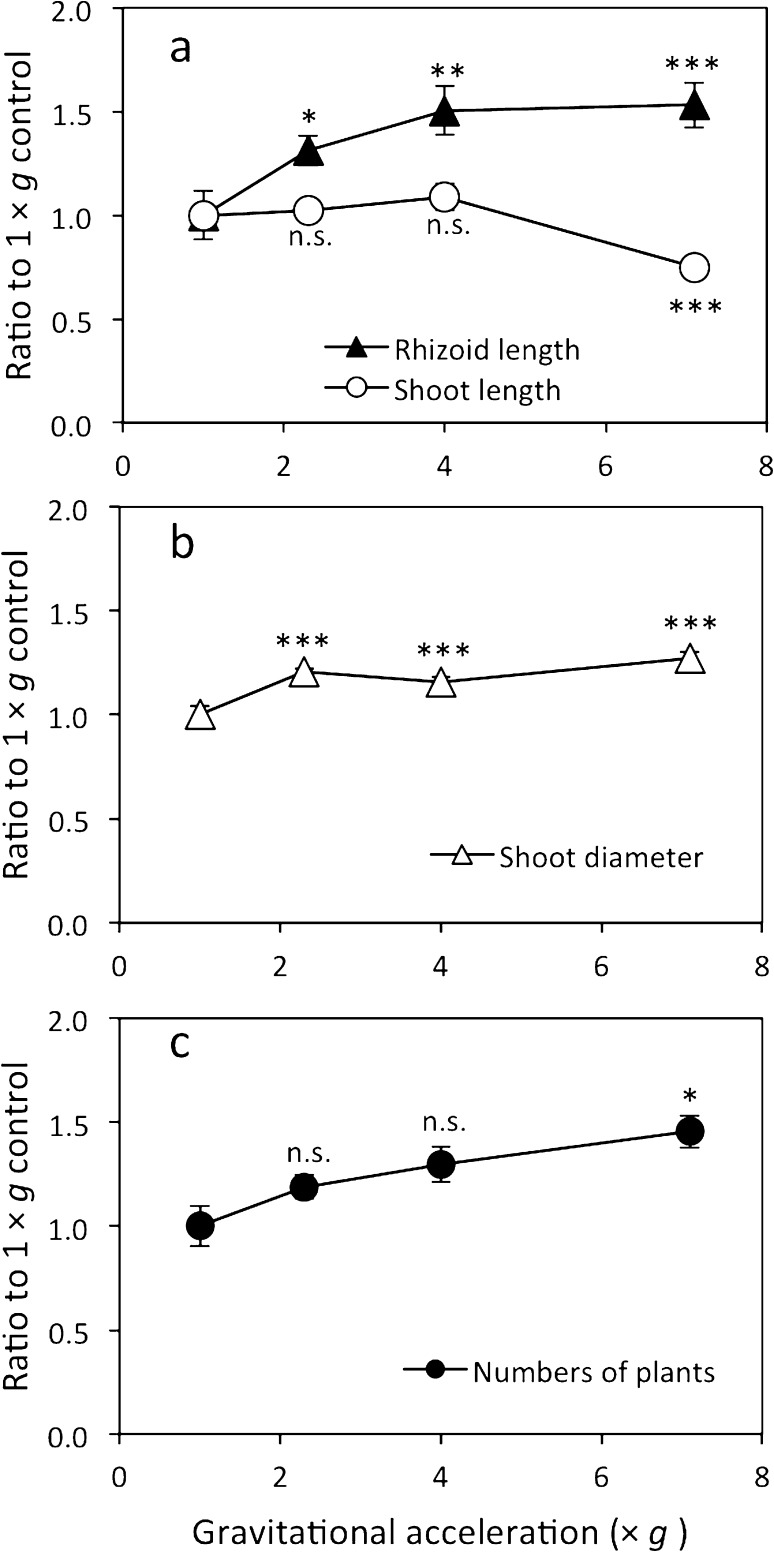
Growth traits of *P. patens* gametophores grown at 2.3, 4.0, and 7.1×*g* for 25 days. The average (± SE) ratios of the parameters to those grown at 1×*g* were shown. **a** Rhizoid length and shoot length (*n* = 10–14), and **b** shoot diameter for each gametophore (*n* = 10–14) and **c** the number of gametophores in a canopy (*n* = 5–6). Differences between 1×*g* and each hypergravity condition (2.3, 4.0 and 7.1×*g*) were analyzed using Welch’s *t*-test, with significant levels of *P < 0.05, **P < 0.01, and ***P < 0.001. n.s. means no significant difference

Hypergravity environments at 2.3, 4.0, and 7.1×*g* for 25 days had opposite effects on the shoot and rhizoid growth of *P. patens* (Fig. 2). Rhizoid length was 53% longer, while shoot length was 25% shorter at 7.1×*g* than at 1×*g* (Fig. 2a). Shoot diameter significantly increased by 16–27% under all hypergravity environments tested (Fig. 2b). Gametophore numbers in the canopy, which was produced during the hypergravity experiment, were significantly higher at 7.1×*g* (by 46%) than that at 1×*g* (Fig. 2c). These results indicate that the long-term and moderate hypergravity environment affected the growth of *P. patens.*

**Fig. 3:**
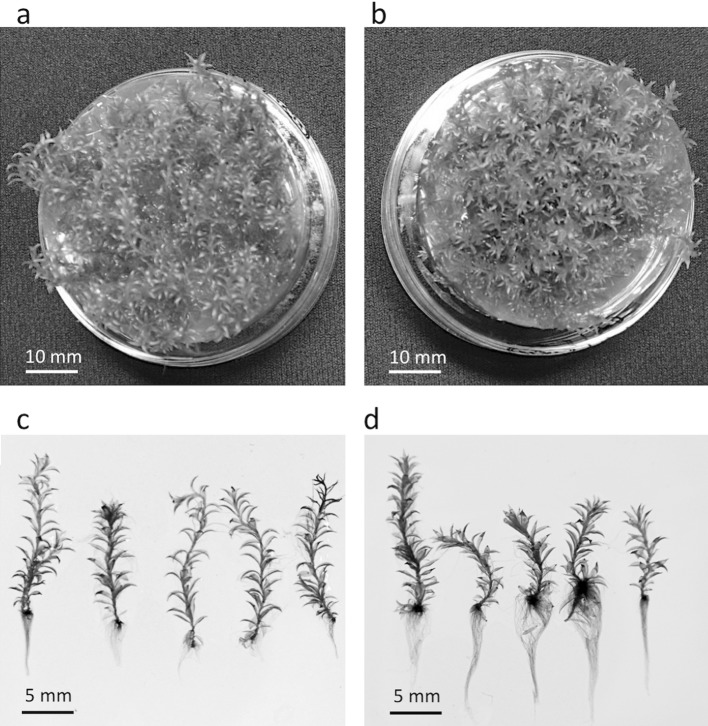
Images of **a**, **b** canopies of *P. patens* and **c**, **d** individual gametophores grown for 8 weeks at 1×*g* (**a**, **c**) or 10×*g* (**b**, **d**)

**Fig. 4:**
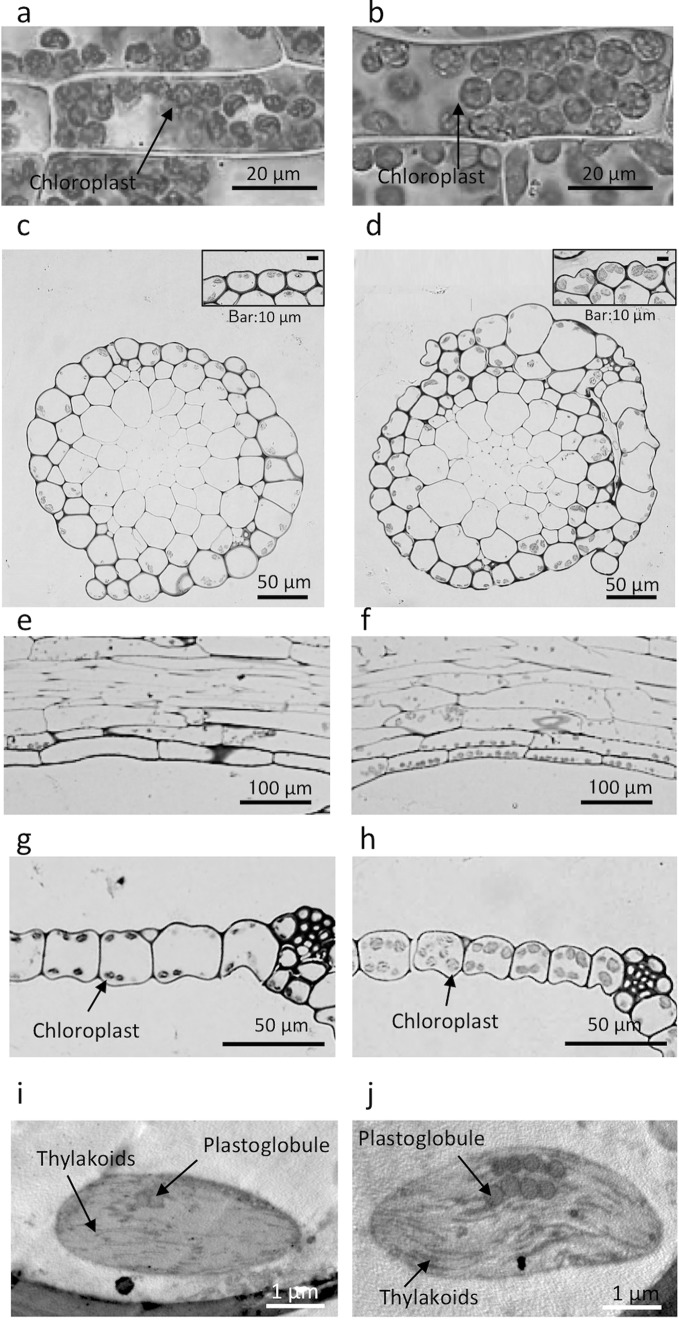
Light and electron micrographs of *P. patens* grown for 8 weeks at (**a**, **c**, **e**, **g**, **i**) 1×*g* or (**b**, **d**, **f**, **h**, **j**) 10×*g*. Micrographs of (**a**, **b**) fresh leaves of *P. patens* at the central part of the lamina, **c**, **d** transverse sections of stems, **e**, **f** longitudinal sections of stems, **g**, **h** transverse sections of lamina, and **i**, **j** chloroplasts in the stem epidermal cells of *P. patens* observed using a transmission electron microscope. Plant samples for (**c**–**j)** were collected at 3 mm from the top of gametophores

The effects of hypergravity on the growth of gametophores of *P. patens* were more prominent when they were grown under the 10×*g* environment for 8 weeks. Shoot length decreased, whereas rhizoid length increased at 10×*g* for individual gametophores (Fig. 3c, d); shoot length was 19% shorter, whereas rhizoid length was 87% longer than those at 1×*g* (Table 1). In accordance with the results for plant length, the dry mass of shoots was 21% lower, whereas that of rhizoids was 154% higher at 10×*g* that at 1×*g*. At the canopy level, gametophore numbers were 85% higher at 10×*g* than that at 1×*g*, with gametophores covering most of the area of the bottoms of Petri dishes under both gravitational conditions (Fig. 3a, b; Table 1). At 10×*g*, canopy-based dry mass significantly increased for shoots and rhizoids by 49 and 331%, respectively. In contrast to the other parameters tested, the numbers of chloroplasts in leaves were similar between 1×*g* and 10×*g* (Table 1; Fig. 4a, b).

**Table 1:** Effects of gravity on the growth of and chloroplast numbers in *P. patens* leaf cells

Traits	1×*g*	10×*g*	*P* value
Gametophore features			
Shoot length (mm)	14.9 ± 2.4	12.1 ± 2.2	***
Rhizoid length (mm)	7.8 ± 1.7	14.6 ± 4.2	***
Shoot dry mass (mg)	0.343 ± 0.056	0.272 ± 0.039	*
Rhizoid dry mass (mg)	0.0240 ± 0.0082	0.0608 ± 0.0172	**
Canopy features			
Shoot dry mass (mg)	32.4 ± 4.7	48.1 ± 7.7	**
Rhizoid dry mass (mg)	2.30 ± 0.73	9.91 ± 3.34	**
Numbers of plants	92.2 ± 4.5	170.7 ± 10.0	***
Numbers of chloroplasts per leaf cell	24.6 ± 4.5	24.5 ± 4.4	n.s.

The length and dry mass of the shoot (leaf + stem) and rhizoid of each gametophore (*n* = 75–76), and the dry mass of shoots and rhizoids in the canopy and numbers of gametophores for each canopy (*n* = 6–7) are shown. The numbers of chloroplasts per each cell were measured for fresh leaves using digitized images (*n* = 54–56). Average ± SD values are shown for all traits. Differences between averaged values were analyzed using Welch’s *t*-test, with significant levels of * *P* < 0.05, ** *P* < 0.01, and *** *P* < 0.001 for the effects of gravity. n.s. means no significant difference

The stem diameters of *P. patens* increased at 10×*g* at the top, but not at the base of the stem (Table 2). Stem cell density estimated from transverse sections (Fig. 4c, d) was not changed at 10×*g*. In the epidermal cells of the stem (Fig. 4e, f), length decreased by 22–26% at 10×*g* at the top and at the base of the stem, while diameter was not changed at 10×*g* at either position. The cell wall thickness of stem epidermal cells was similar between the 10×*g* and 1×*g* conditions (Table 2). Leaf cells of *P. patens* were single layered (Fig. 4g, h). Leaf length significantly decreased at 10×*g* by 14–17% (Table 2). Leaf lamina cell diameter at the top of the stem decreased by 27% at 10×*g*. Lamina thicknesses and cell numbers were not changed at 10×*g*. The cell wall thickness of leaves at 10×*g* significantly decreased by 13–19%. No significant alteration in the thylakoid structure of chloroplasts in leaf cells was observed at 10×*g* (Fig. 4i, j). Chloroplasts were discoid or spindle-shaped, contained a well-developed lamellar system, and had few plastoglobuli.

**Table 2:** Effects of gravity on anatomical traits of *P. patens*

	Top of the stem			Base of the stem		
Traits	1×*g*	10×*g*	*P* value	1×*g*	10×*g*	*P* value
Stem						
Diameter (mm)	0.19 ± 0.02	0.23 ± 0.03	*	0.21 ± 0.03^n.s.^	0.23 ± 0.04^n.s.^	n.s.
Cell density (0.1 mm^−2^)	206 ± 9	225 ± 32	n.s.	226 ± 16^#^	257 ± 29^n.s.^	n.s.
Epidermal cells						
Length (μm)	163 ± 52	121 ± 23	**	157 ± 39^n.s.^	123 ± 22^n.s.^	**
Diameter (μm)	21.2 ± 4.9	20.4 ± 4.9	n.s.	21.1 ± 5.4^n.s.^	21.4 ± 4.1^n.s.^	n.s.
Cell wall thickness (μm)	1.08 ± 0.19	1.08 ± 0.19	n.s.	1.07 ± 0.23^n.s.^	1.13 ± 0.2^n.s.^	n.s.
Leaf						
Length (mm)	2.29 ± 0.17	1.98 ± 0.25	***	2.44 ± 0.32^n.s.^	2.03 ± 0.29^n.s.^	***
Lamina cell diameter (μm)	32.0 ± 3.0	23.2 ± 4.9	***	21.2 ± 2.6^##^	20.6 ± 3.1^n.s.^	n.s.
Lamina thickness (μm)	26.1 ± 6.4	23.9 ± 7.9	n.s.	23.6 ± 4.7^n.s.^	22.1 ± 0.7^n.s.^	n.s.
Lamina cell number	10.0 ± 3.3	11.6 ± 3.6	n.s.	14.4 ± 1.3^#^	14.7 ± 0.6^n.s.^	n.s.
Cell wall thickness (μm)	0.26 ± 0.05	0.21 ± 0.04	***	0.25 ± 0.03^n.s.^	0.22 ± 0.04^n.s.^	***

Stem and leaf traits were measured at the top (3 mm from the top) and base (3 mm from the base) of stems, respectively. Average ± SD values are shown for all traits. Fresh gametophores with averaged sizes were used to measure stem diameters (*n* = 10–15) and leaf lengths (*n* = 20). The cell density of stems was measured from transverse sections of stems (*n* = 4–6). The averaged length and diameter (*n* = 20–22) and cell wall thickness (*n* = 45) of the epidermal cells of stems were measured from longitudinal and transverse sections of gametophores, respectively. The lamina thickness (*n* = 3–11), lamina cell number from the costa to lamina margin (*n* = 3–6), and cell wall thickness of leaf cells from the 1st to 5th cell from the margin of the lamina (*n* = 60) were measured in a cross section of the leaf lamina. Differences were analyzed using Welch’s *t*-test, with significant levels of * *P* < 0.05, ** *P* < 0.01, and *** *P* < 0.001 for the effects of gravity, and # *P* < 0.05 and ## *P* < 0.001 for the effects of stem position (top or base). n.s. means no significant difference

**Fig. 5:**
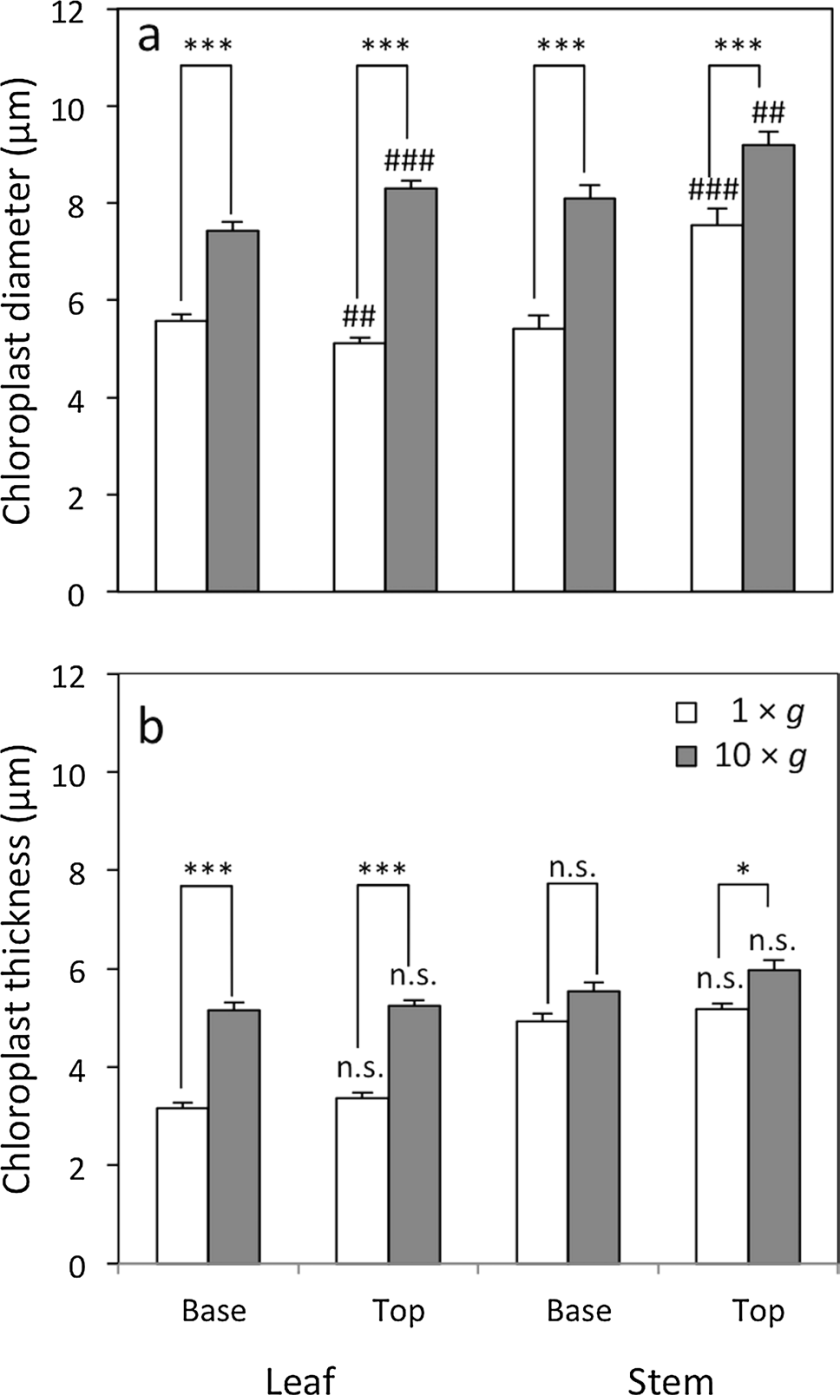
Effects of hypergravity on chloroplast sizes of leaves and stems of *P. patens* at the top (3 mm from the top) and base (3 mm from the base) of stems grown at 1×*g* (*open bars*) or 10×*g* (closed bars). **a** The diameters (major axis) and **b** thicknesses (minor axis) of chloroplasts were measured for 20 chloroplasts from the 1st to 10th cell from the costa of a leaf from 3 gametophores (*n* = 60), and for 15 chloroplasts from the epidermal cells of the stems of 3 gametophores (*n* = 45). Average ± SE values are shown. Differences were analyzed using Welch’s *t*-test, with significant levels of *P < 0.05 and ***P < 0.001 for the effects of gravity, and ^##^ *P* < 0.01 and ^###^ *P* < 0.001 for the effects of the stem position (top or base). n.s. means no significant difference

**Fig. 6:**
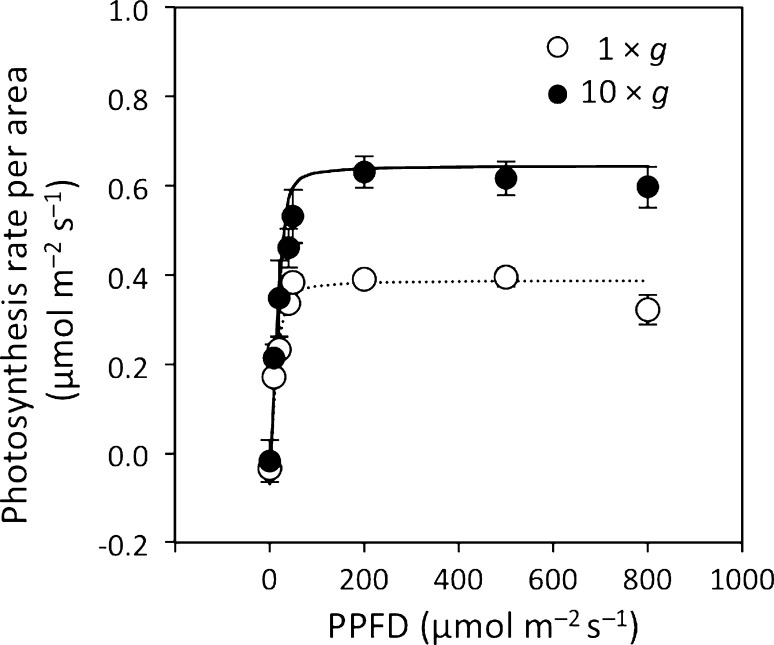
Canopy-based photosynthesis rate per area of *P. patens* against changing photosynthetic photon flux density (PPFD), which was fit using the light response curve model by Ögren and Evans (1993). Measurements were performed at a plant temperature of 25°C, flow rate of 300–350 ml min^−1^, relative humidity of 90%, and ambient CO_2_ concentration of 400 µmol mol^−1^ (*n* = 3-4). PPFD was gradually changed from 0 to 800 µmol m^−2^ s^−1^ at a fully turgid mass. Average ± SE values are shown. Open and closed symbols represent 1×*g* and 10×*g*, respectively

In leaf cells of the base and top of the stems, the diameter and thickness of chloroplasts at 10 × *g* significantly increased by 33–63% (Fig. 5). Similarly, chloroplast sizes in stem epidermal cells at 10×*g* significantly increased by 15–50%, except for the thickness at the base of the stem. The area-based photosynthesis rate was higher at 10×*g* than at 1×*g* (Fig. 6). The area-based photosynthesis rate was light-saturated at 200 µmol m^−2^ s^−1^ for both 10×*g* and 1×*g*. Light-saturated photosynthesis rates calculated from light response curves were 0.416 (0.060) μmol m^−2^ s^−1^ and 0.676 (0.087) μmol m^−2^ s^−1^ at 1×*g* and 10×*g*, respectively, and these values were significantly different (Welch’s *t*-test, *P* < 0.01).

## Discussion

The significant increase observed in canopy-based plant growth at 10×*g* (Table 1) is a novel result for plant responses to hypergravity, which contrasts with our hypothesis. We achieved enhanced rhizoid growth by hypergravity in the present study; on the other hand, previous studies reported a decline in root length for pea sprouts subjected to 140–1054×*g* (Waldron and Brett 1990). Although the underlying mechanisms currently remain unknown, the enhancements observed in rhizoid growth and plant numbers by hypergravity (Table 1; Fig. 2) may be unique features for mosses and may be affected by some phytohormones such as auxin and/or cytokinin. The increase in plant numbers may be affected by higher numbers of buds differentiating from the caulonema, with their differentiation being affected by auxin (Reski 1998). Tamaoki et al. (2009, 2011) indicated that the endogenous accumulation of auxin was enhanced in the inflorescence stems of *A. thaliana* in a 300×*g* environment.

Novel results were also obtained for chloroplast sizes in the leaves and stems of gametophores grown at 10×*g* (Fig. 4a-h); the diameters (major axis) and thicknesses (minor axis) of chloroplasts at 10×*g* increased by 15–63% (Fig. 5). In angiosperms, an increase in the surface area of chloroplasts for CO_2_ diffusion has been shown to enhance photosynthesis in leaves (Terashima et al. 2006, 2011). The number of chloroplasts per leaf cell was not affected by hypergravity in *P. patens* (Table 1); therefore, the enlargement observed in chloroplasts may increase their surface area exposed to air, indicating that the hypergravity environment enhances CO_2_ diffusion. This enhancement in CO_2_ diffusion may partly cause an increase in area-based photosynthesis in the leaves of *P. patens* (Fig. 6). Plant growth hormones such as auxins may affect chloroplast sizes under hypergravity conditions because the homeodomain-leucine zipper I gene, *Pphb7*, which is regulated by exogenous auxin, affects increases in the sizes of chloroplasts (Sakakibara et al. 2003). Chloroplasts in angiosperms are typically ~5 μm in diameter with a thickness of ~2.5 μm (Staehelin 2003); chloroplasts in the leaves of *P. patens*, 5.1–8.3 μm in diameter with a thickness of 3.2–5.2 μm (Fig. 5), are larger than those of angiosperms, which may contribute to increases in the surface area of chloroplasts for CO_2_ diffusion. In the evolutionary history of land plants, the earliest land plants such as bryophytes may have acquired the ability to regulate CO_2_ diffusion by adjusting the size of chloroplasts. In ferns and angiosperms, photosynthesis and CO_2_ diffusion may be effectively regulated via stomatal control and/or the development of mesophyll tissues (Carriquí et al. 2015; Tosens et al. 2016).

A thickness of cell wall is considered to be one of the main factors reducing CO_2_ diffusional conductance in bryophytes (Hanson et al. 2014). Hypergravity-induced reductions in cell wall thickness in the leaves of *P. patens* (Table 2) suggest that hypergravity did not decrease CO_2_ diffusion through cell walls, which is in contrast to our hypothesis that hypergravity increases cell wall thickness and, thus, reduces the photosynthesis rate; we obtained an increase in the area-based photosynthesis rate by hypergravity (Fig. 6). The cell wall thickness of the leaves of *P. patens*, 0.21–0.26 μm, is within the lowest range of cell wall thicknesses for angiosperms (0.1–0.6 μm) and ferns (0.2–0.8 μm) (Tosens et al. 2016); this may contribute to less resistance to CO_2_ diffusion in *P. patens* than in angiosperms.

The increase observed in the area-based photosynthesis rate under hypergravity conditions (Fig. 6) is strongly affected by greater amounts of photosynthesis tissues on an area basis, which is mainly caused by larger canopy-based plant numbers (Table 1). The stimulation of the convection of gasses under the hypergravity condition, which may enhance CO_2_ transport to the leaves and increase the photosynthesis rate (Hirai and Kitaya 2009), is less possible in the present study because of the small height of the Petri dishes (3 cm) used for plant growth. As previously discussed, the increases observed in chloroplast sizes may partly affect those in the area-based photosynthesis rate under hypergravity conditions via enhanced CO_2_ diffusion in the leaves of *P. patens*. Additionally, the decrease in cell wall thickness in leaves (Table 2) may also enhance CO_2_ diffusion and thus contribute partly to the increase in area-based photosynthesis rate. Waite and Sack (2010) reported a strong positive relationship between area-based photosynthesis rates and canopy densities for 10 field-grown Hawaiian moss species, which indicates that thick and dense photosynthesis tissues involve high area-based photosynthesis rates for the canopies of moss species. The light-saturated area-based photosynthesis rates of *P. patens*, 0.41–0.69 μmol m^−2^ s^−1^, are near the lowest values for 10 field-grown Hawaiian moss species, 0.59–1.41 μmol m^−2^ s^−1^ (Waite and Sack 2010).

Previous studies reported reductions in shoot elongation by short-term strong hypergravity (more than 30×*g*) under dark conditions for the sprouts of various angiosperms including pea, cress, maize, azuki bean, and *A. thaliana* (Hoson et al. 1996; Soga et al. 1999a, b, 2001; Waldron and Brett 1990). The reductions induced in the shoot growth of individual gametophores of *P. patens* by long-term moderate hypergravity under light conditions in the present study (Fig. 2a; Table 1) support the previous findings described above. The suppression of shoot growth by hypergravity has been attributed to modifications in cell walls including an increase in thickness and a decrease in elasticity caused by the accumulation of xyloglucans (Soga et al. 1999b) and/or an increase in secondary cell wall lignification (Tamaoki et al. 2006). Alterations in the orientation of cortical microtubules during the hypergravity condition, e.g., a decrease in cells with transverse microtubules and an increase in cells with longitudinal microtubules, involve the inhibition of elongation growth and enhancement of lateral growth (Soga et al. 2006). In the present study, an increase was not observed in cell wall thickness in the shoots or leaves of *P. patens* grown under hypergravity conditions (Table 2); however, alterations in the cell wall compositions or molecular masses of cell wall polysaccharides are still possible (Soga et al. 1999b). Specific variations exist in cell types and cell wall compositions, with mosses lacking true secondary cell walls unlike other plant taxa, but containing polysaccharides such as xyloglucan, xylan, and pectin in their primary cell walls, similar to other plant taxa (Popper 2008). The decrease observed in cell wall thickness under hypergravity conditions in the leaves of *P. patens* (Table 2) suggests that the development of primary cell walls in the leaves of *P. patens* is inhibited by hypergravity.

In the present study, reductions in the cell length of the epidermal cells of stems (Table 2) may involve decreases in the elongation of the shoots of individual gametophores (Table 1) because epidermal cell layers in angiosperms influence the rate of organ elongation (Kutschera and Niklas 2007; Savaldi-Goldstein and Chory 2008). Similarly, the reduction noted in leaf length (Table 2) was affected by decreases in cell sizes because cell numbers were not altered by hypergravity (Table 2).

## Conclusions

We herein demonstrated some unique features for the responses of the growth and anatomy of *P. patens* to hypergravity conditions. Increases in the canopy-based growth of shoots mainly caused by greater plant numbers, and the larger chloroplast sizes observed under hypergravity conditions are all novel results, and may be affected by alterations in the functions of phytohormones. The increases observed in plant numbers and, partly, chloroplast sizes involved a higher area-based photosynthesis rate by *P. patens* under hypergravity conditions. The absence of an increase in cell wall thickness in stem cells or even a decrease in leaf cells under hypergravity conditions contradicts our hypothesis and previous findings for angiosperms; since the growth forms of mosses are groups of a large number of individual gametophores, an increase in plant density rather than in the cell toughness of individual gametophores may be more effective for adapting to a hypergravity environment.
