# Correction to: A hypergravity environment increases chloroplast size, photosynthesis, and plant growth in the moss *Physcomitrella patens*

**DOI:** 10.1007/s10265-018-1054-5

**Published:** 2018-07-18

**Authors:** Kaori Takemura, Hiroyuki Kamachi, Atsushi Kume, Tomomichi Fujita, Ichirou Karahara, Yuko T. Hanba

**Affiliations:** 1 Department of Applied Biology, Kyoto Institute of Technology, Matsugasaki, Sakyo-ku, Kyoto, 606-8585 Japan; 2 Graduate School of Science and Engineering, University of Toyama, 3190 Gofuku, Toyama, 930-8555 Japan; 3 Faculty of Agriculture, Kyushu University, 6-10-1 Hakozaki, Higashi-ku, Fukuoka, 812-8581 Japan; 4 Faculty of Science, Hokkaido University, Kita-ku, Sapporo, 060-0810 Japan

## Correction to: J Plant Res (2017) 130:181–192 10.1007/s10265-016-0879-z

The article “A hypergravity environment increases chloroplast size, photosynthesis, and plant growth in the moss *Physcomitrella patens*”, written by Kaori Takemura, Hiroyuki Kamachi, Atsushi Kume, Tomomichi Fujita, Ichirou Karahara and Yuko T. Hanba, was originally published electronically on the publisher’s internet portal (currently SpringerLink) on 28 November 2016 without open access.

With the author(s)’ decision to opt for Open Choice the copyright of the article changed on 16 July 2018 to © The Author(s) 2018 and the article is forthwith distributed under the terms of the Creative Commons Attribution 4.0 International License (http://creativecommons.org/licenses/by/4.0/), which permits use, duplication, adaptation, distribution and reproduction in any medium or format, as long as you give appropriate credit to the original author(s) and the source, provide a link to the Creative Commons license and indicate if changes were made.

